# Diphtheria

**Published:** 1876-12

**Authors:** 


BARK VALLEY, FULTON CO., PA., Nov. 16, 1876.

MR. EDITOR—DEAR SIR: Having been quite interested in the articles in your valuable journal on "Diphtheria," allow me to suggest as a local remedy peppermint. I use the (candy) drops; let them dissolve slowly in the mouth, as many as required to keep up the impression of cold at each inspiration. I speak from personal experience, as well as several years' trial in practice. The easy administration, especially to children; the instantaneous and marked relief which it affords, make me believe it a valuable adjunct to our local remedies; in fact, I use it in all diseases of the throat where there is a hyperæmic condition, and through an epidemic of scarlatina, and in all cases with equally good results.

W. L. MCKIBBIN, M.D.

The above short correspondence is taken from November

No. of *Philadelphia Medical Times*, and in connection with the subject, we would suggest the propriety of using remedies calculated to produce more decided action, in case of failure with the peppermint candy. The question naturally presents itself, are local remedies sufficient to meet the indications in severe cases of diphtheria? Whether this should be answered negatively or not, certain it is that antiseptics and defibrinating antiphogistics seem to be called for. Though the disease be purely a local inflammation, as ordinary croup and tonsilitis, elective or general means, as well as local, are often required in order to control it. Carbolic acid may be made to answer the purposes of local alterative and antiseptic. Much depends, however, upon the manner of using this acid for its topical effects. If very much diluted, no benefit from its application need be expected; and, indeed, when used in the strength of eight or ten grains to the ounce of water, the excitement seems to be increased. The acid in full strength, warmed and applied with a soft brush to the whole surface diseased, so far as it can be reached, not only aids very much in arresting the pseudo-membranous formation, but the inflammatory action upon which it depends. Unfortunately, however, this mode is not applicable to the trachea and bronchial air passages. Is the acid sufficiently volatile to be inhaled, and would it be tolerated in sufficient quantity?

